# A Rare Case of Adrenal Carcinoma With Isolated Hypercortisolism Mimicking Hyperaldosteronism

**DOI:** 10.7759/cureus.61481

**Published:** 2024-06-01

**Authors:** Sai Rakshith Gaddameedi, Axle D Untalan, Malay Rathod, Phani Bhavana Cherukuri, Vandana Bandari, Manjula Ashok, Doantrang Du

**Affiliations:** 1 Internal Medicine, Rutgers Health/Monmouth Medical Center, Long Branch, USA; 2 Medicine, Rutgers Health/Monmouth Medical Center, Long Branch, USA; 3 Internal Medicine, Bayhealth Medical Center, Dover, USA; 4 Nephrology, Rutgers Health/Monmouth Medical Center, Long Branch, USA

**Keywords:** adrenal cancer, adrenalectomy, adrenal mass, hyperaldosteronism, hypercortisolism, adrenal cortical carcinoma

## Abstract

We report a case of a 22-year-old female with pedal edema, hypokalemia, and hypertension. On suspicion of hyperaldosteronism, further workup was pursued, which only revealed a low serum adrenocorticotropic hormone (ACTH) and an inappropriately normal cortisol level after a 1-mg dexamethasone suppression test, suggestive of primary hypercortisolism. CT of the chest, abdomen, and pelvis revealed a left adrenal mass. Based on the clinical findings and biochemical abnormalities, we were expecting this tumor to be aldosterone-secreting, but both serum aldosterone and renin levels were normal in our patient. Eventual surgical resection confirmed initial suspicions of malignancy, as it was found to be adrenal cortical carcinoma. This case highlights the unusual presentation of this rare but aggressive endocrinologic neoplasm and the importance of its prompt diagnosis and treatment.

## Introduction

Adrenal carcinomas are uncommon malignancies arising from the adrenal cortex, accounting for only 0.2% of all cancers. While these tumors often present with a myriad of clinical manifestations due to excessive hormone production, isolated hypercortisolism as the primary manifestation is a rare occurrence [1]. Hyperaldosteronism, typically associated with aldosterone-producing adenomas or bilateral adrenal hyperplasia, is a well-recognized cause of secondary hypertension [2]. However, adrenal carcinomas, with their propensity for autonomous cortisol secretion, can present with a clinical profile that closely resembles primary aldosteronism. This case report details an exceptional case of adrenal carcinoma characterized by isolated hypercortisolism mimicking the clinical and biochemical features of hyperaldosteronism. Understanding the nuances of this unusual presentation is crucial for timely diagnosis and management. Given the rarity of adrenal cortical carcinoma and its aggressive nature, prompt identification and surgical resection remain essential for effective management and improved outcomes in such cases [3].

## Case presentation

We present a case of a 22-year-old female with anxiety, insomnia, urge incontinence, and irritable bowel syndrome, who presented to the ED with a complaint of bilateral ankle swelling for the last two days and reported severe migraine-like headaches in the previous month. She denied loss of consciousness, visual changes, numbness, focal neurological deficits, slurred speech, weight gain, easy bruising, muscle weakness, menstrual irregularities, or any other associated symptoms. Home blood pressure readings were elevated, with the highest at 180/92 mmHg. In the ED, her blood pressure was 182/125 mmHg, her heart rate was 78 bpm, and she was saturating well on room air. A physical exam revealed a flow murmur at the left sternal border and grade 1 pitting edema in the bilateral ankles. Initial labs were significant for potassium 2.1 mmol/L and B-type natriuretic peptide (BNP) 177 pg/mL (Table 1). She was admitted for the management of hypertension with etiology under investigation. Her family history was significant for early-onset hypertension in her father.

**Table 1:** Pertinent laboratory results with reference values, initial workup BUN: blood urea nitrogen; ALT: alanine transaminase; AST: aspartate aminotransferase; ALP: alkaline phosphatase; BNP: B-type natriuretic peptide; MCV: mean corpuscular volume; MCH: mean corpuscular hemoglobin; MCHC: mean corpuscular haemoglobin concentration

Laboratory test	Patient’s result	Reference value
Serum sodium	142 mmol/L	135 – 145 mmol/L
Serum potassium	2.1 mmol/L	3.5 – 5.2 mmol/L
Serum chloride	102 mmol/L	99 – 109 mmol/L
Serum CO2	32 mmol/L	24 -35 mmol/L
Serum BUN	10 mg/dL	5 – 21 mg/dL
Serum creatinine	0.56 mg/dL	0.4 – 1.1 mg/dL
Serum ALT	34 U/L	10 – 43 U/L
Serum AST	32 U/L	13 – 41 U/L
Serum ALP	79 U/L	42 – 119 U/L
Serum total protein	6.6 g/dL	6.4 – 8.3 g/dL
Serum albumin	4.2 g/dL	3.5 – 5.0 g/dL
Serum calcium	7.7 mg/dL	8.3 – 10.2 mg/dL
Serum magnesium	2.3 mg/dL	1.5 – 2.5 mg/dL
Serum glucose	131 mg/dL	70 – 110 mg/dL
Serum BNP	177 pg/mL	<=100 pg/mL
Hemoglobin	12.4 g/dL	12.0 – 16.0 g/dL
Hematocrit	33.8%	38.0 – 47.0%
White blood cells	8.40 10^3^/uL	4.5 – 11.0 10^3^/uL
Red blood cells	4.11 10^6^/uL	4.20 – 5. 4 10^6^/uL
MCV	82.2 fL	81.0 – 98.0 fL
MCH	30.2 pg	27.0 – 32.0 pg
MCHC	36.7 g/dL	32.0 – 36.0 g/dL
Platelets	266 10^3^/uL	140 – 450 10^3^/uL

**Figure 1:**
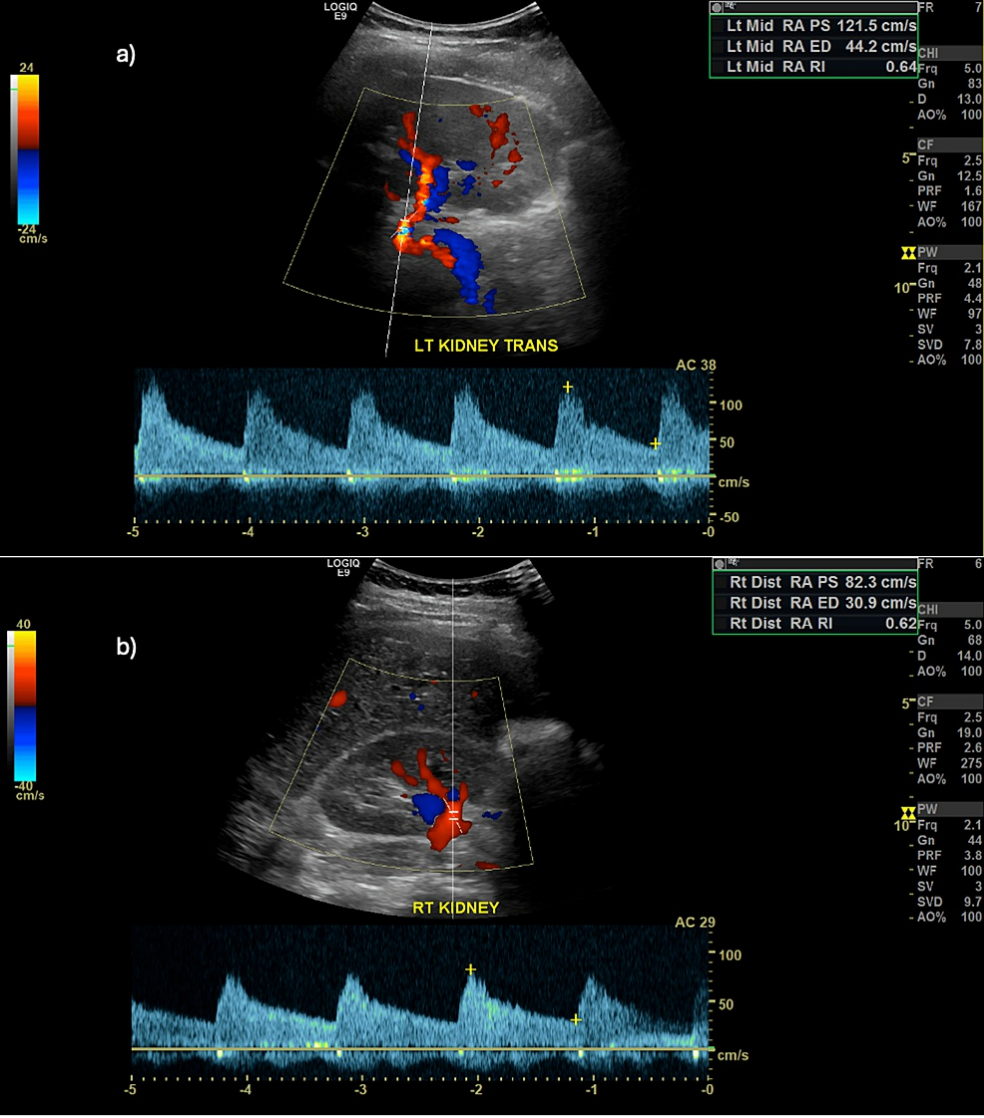
Renal duplex ultrasound a) Good arterial flow in the left mid-renal artery b) Good arterial flow in the right distal renal artery

A battery of investigations was performed to investigate new-onset hypertension. Renal duplex ultrasound was negative for renal artery stenosis (Figure 1) and an echocardiogram showed an ejection fraction of 60-65% with normal left ventricular function. A basic metabolic panel was performed every six hours to replete potassium as needed. She was started on Spironolactone 25 mg daily and Labetalol 100 mg daily for the management of hypertension. Labetalol was replaced with Nifedipine 60 mg twice daily the following day to fine-tune the blood pressure control.

Serum and urine metanephrines and normetanephrine, along with catecholamines, were within the normal range. Dehydroepiandrosterone (DHEA) was low at 73.4 ug/dl. Serum aldosterone and renin were normal. Cortisol post-1 mg dexamethasone suppression test was 3.73 ug/dL, indicating no suppression and hypercortisolism. ACTH before morning cortisol was low at 5 pg/ml, suggesting ACTH-independent Cushing disease, most likely from an adrenal mass secreting excessive cortisol. Testosterone levels were also within the normal range (Table 2). Treatment adjustments included increasing Spironolactone to 50 mg twice daily and reducing Nifedipine to 30 mg twice daily. Nifedipine was eventually replaced with Doxazosin 1 mg twice daily, and both Spironolactone and Doxazosin doses were further increased to 100 mg and 2 mg twice daily, respectively.

**Table 2:** Pertinent laboratory results with reference values, further workup DHEA: dehydroepiandrosterone; ACTH: adrenocorticotropic hormone

Laboratory test	Patient’s result	Reference value
Plasma metanephrines	16.3 pg/mL	0 – 88.0 pg/mL
Plasma normetanephrines	31.1 pg/mL	0 – 210.1 pg/mL
Plasma epinephrine	31 pg/mL	0 – 62.0 pg/mL
Plasma norepinephrine	44 pg/mL	0 – 874.0 pg/mL
Plasma dopamine	<30 pg/mL	0 – 48.0 pg/mL
24-hour urine metanephrine	55 ug/24 hr	36.0 – 209.0 ug/24 hr
24-hour urine normetanephrine	233 ug/24 hr	95.0 – 449.0 ug/24 hr
Serum DHEA	73.4 ug/dL	110.0 – 431.7 ug/dL
Serum aldosterone	28.5 ng/dL	0 – 30.0 ng/dL
Plasma renin	0.291 ng/mL/hr	0.167 – 5.380 ng/mL/hr
Cortisol post-1 mg dexamethasone suppression test	3.73 ug/dL	1.0 – 75.0 ug/dL
Plasma ACTH	5.0 pg/mL	7.2 – 63.3 pg/mL
Serum testosterone, total	21.2 ng/dL	10.0 – 55.0 ng/dL
Serum testosterone, free	0.21 ng/dL	0.10 – 0.85 ng/dL
Serum testosterone, free %	0.99%	0.50 – 2.80%

**Figure 2:**
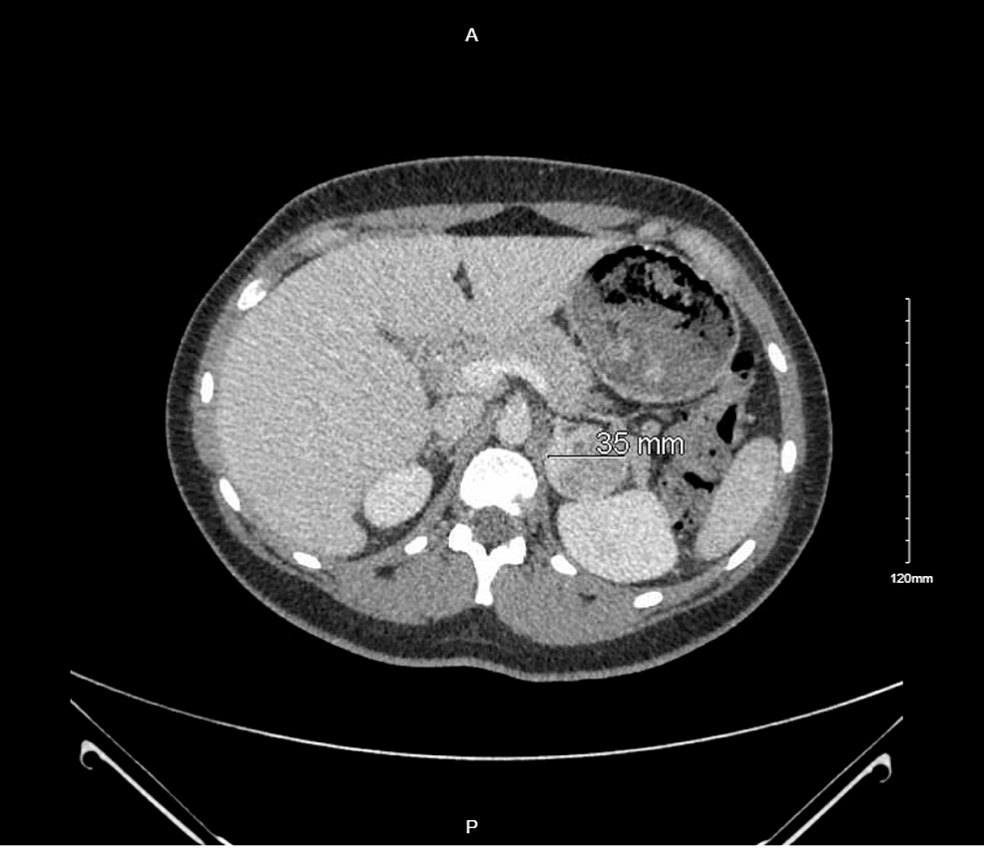
CT abdomen with and without contrast showing a left adrenal heterogeneously enhancing nodule measuring 35 x 68 x 35 mm

**Figure 3:**
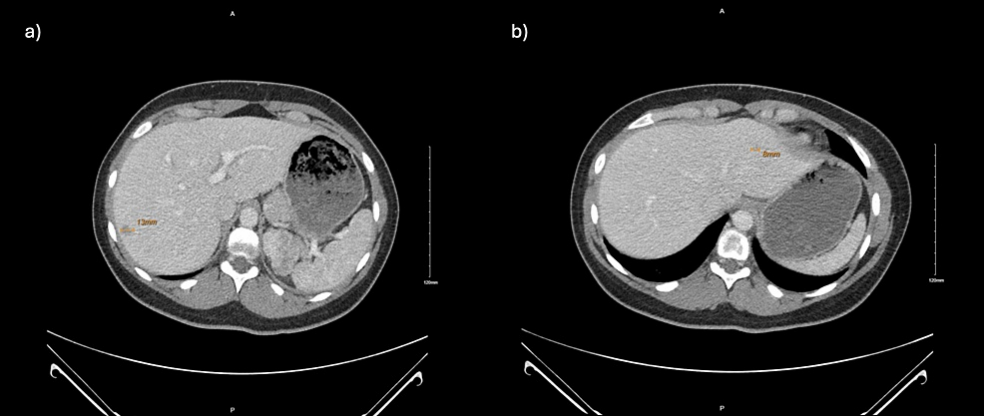
CT abdomen with and without contrast showing two hepatic lesions measuring 13 mm (a) and 8 mm (b)

**Figure 4:**
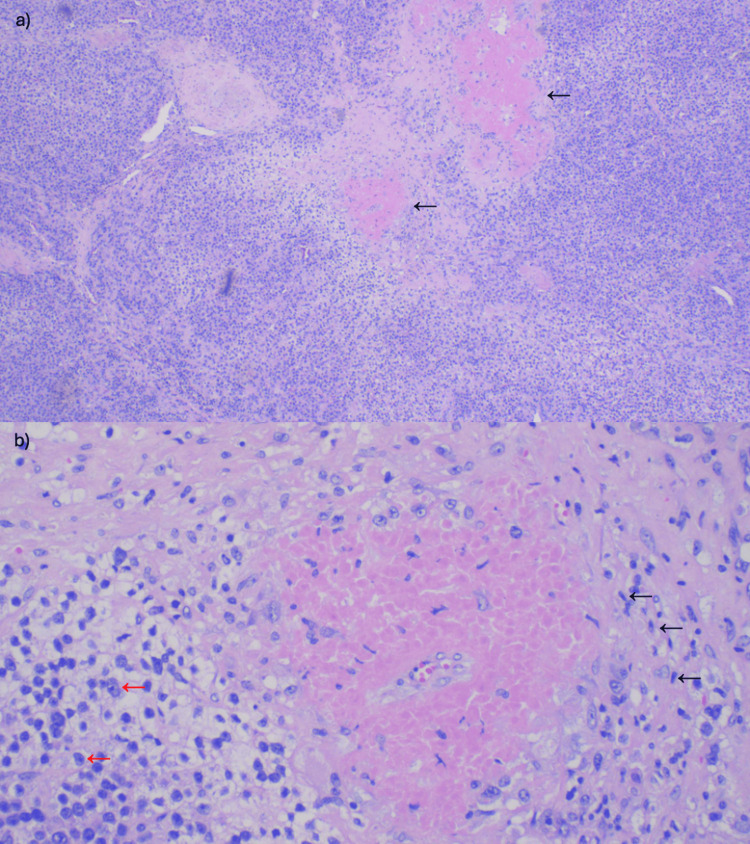
Left adrenal gland histology a) HE 4x: tumor cells resembling cortical cells of zona glomerulosa with a high N/C ratio and areas demonstrating polygonal cells with abundant eosinophilic or clear cytoplasm (black arrows) b) HE 20x: overt nuclear pleomorphism (black arrows) and vesicular nuclei with prominent nucleoli (red arrows) N/C ratio: nuclear/cytoplasmic ratio

**Figure 5:**
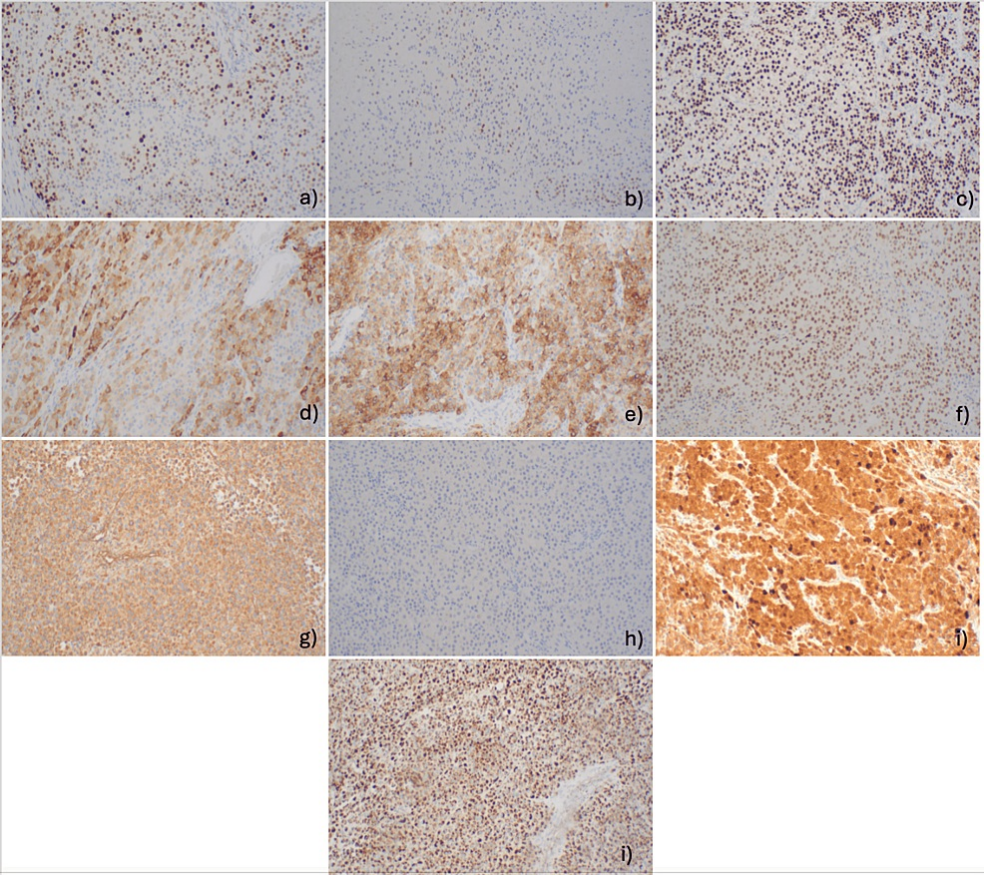
Left adrenal gland biopsy special studies a) Ki-67 expression of approximately 30%, b) P53 expression of approximately 30%, c) SF-1 positive, d) inhibin positive, e) synaptophysin positive, f) GATA-3 positive, g) vimentin-positive, h) Pax8 negative, i) beta-catenin with a nuclear, cytoplasmic, and focal membranous staining pattern, j) SDH-B stain retained

Due to the above findings, a CT scan of the abdomen was performed for further investigation, which revealed a 35 mm x 68 mm x 35 mm heterogeneous left adrenal mass (Figure 2) with precontrast, portal venous phase, and delayed phase attenuation values of 40 HU, 65 HU, and 63 HU, respectively, with two hepatic lesions measuring 13 mm and 8 mm (Figure 3). These findings were confirmed on MRI. CT scan of the chest and pelvis did not show any evidence of metastasis. A biopsy of the liver lesion revealed benign-appearing hepatocytes. Due to high suspicion of malignancy, she underwent a left adrenalectomy. Hydrocortisone was given to prevent postoperative adrenal insufficiency. A tissue biopsy revealed adrenal cortical carcinoma (Figures 4-5). Mitotane, along with surveillance with a CT scan, was initiated due to the high risk of recurrence, as supported by a high proliferative index with Ki-67 >30%. She is following up with an endocrinologist and oncologist regularly for surveillance.

## Discussion

Unilateral adrenal tumors are common and are found incidentally on imaging. The majority of these are benign and nonfunctioning. Adrenocortical carcinoma (ACC) is a rare endocrine malignancy, occurring in only 0.5-2 cases per 1 million populations per year, and has a generally unfavorable prognosis, with a 5-year survival rate of 45-60 percent in those with early-stage disease and 10-25% in those with advanced stage disease. It can be found in any age group, but its incidence is higher in children under five and patients in their forties or fifties. It tends to affect females more, with a female-to-male ratio of 1.5 to 2.5:1 [3]. Most cases occur sporadically, but they can occur in association with hereditary cancer syndromes such as Li-Fraumeni syndrome, Beckwith-Wiedemann syndrome, and multiple endocrine neoplasia type 1 [3]. 

Patients with ACC can present asymptomatically, with the tumor being found incidentally on imaging. Some can present with nonspecific gastrointestinal symptoms, such as abdominal pain or fullness, which are due to the effects of local tumor growth. In about 40-60% of patients, signs and symptoms of hormone overproduction can be seen. Glucocorticoid excess typically causes Cushing syndrome [3]. Our patient’s ACTH level was low at 5.0 pg/mL, and her cortisol level was inappropriately average after a 1-mg dexamethasone suppression test, suggestive of primary hypercortisolism. Interestingly, she did not present with the classic signs and symptoms of cortisol excess such as moon facies, buffalo hump, central obesity, abdominal striae, thin hair, thin skin with easy bruising, or weight gain. Her main presenting signs were hypertension and hypokalemia, which we suspected were from an excess of aldosterone, but this level was normal on further workup. The elevated cortisol levels in ACC can lead to a glucocorticoid-mediated mineralocorticoid receptor activation due to the overwhelming of the renal 11 beta-hydroxysteroid dehydrogenase type 2 enzyme, which converts cortisol to cortisone. This creates an apparent mineralocorticoid excess, leading to hypertension and hypokalemia [4]. Patients can also have androgen excess, causing virilization and menstrual irregularities in female patients. In male patients, on the other hand, the androgen excess can lead to peripheral conversion of androgens to estrogen, causing gynecomastia and testicular atrophy, although the tumor itself can also produce estrogen [5].

Evaluation for hormonal secretion includes the following tests: fasting blood glucose, serum potassium, cortisol, corticotropin (ACTH), 24-hour urinary free cortisol, dexamethasone suppression test, dehydroepiandrosterone sulfate (DHEAS), androstenedione, testosterone, 17-hydroxyprogesterone, and serum estradiol [6]. It is also essential to measure plasma or urinary metanephrines and catecholamines to rule out pheochromocytoma. For those presenting with hypertension and/or hypokalemia, plasma aldosterone and renin should be measured [6]. Other than the low ACTH and inappropriately normal cortisol, the above levels were within normal limits for our patient.

Most hormone-secreting ACCs present with Cushing’s syndrome. Patients presenting with hypertension and hypokalemia without signs and symptoms of Cushing’s syndrome are rare. In one case reported in Nepal, a patient with ACC presented with hypertension and hypokalemia, but his tumor was found to be aldosterone-secreting [7]. Another case report described a patient with an advanced-stage cortisol-secreting ACC who initially presented similarly to our patient, but was later on found to have had vague symptoms of Cushing’s syndrome in the past upon further history-taking [8].

On presentation, ACC tumors are typically large, with a diameter usually more than 4 cm. Abdominal computed tomography (CT) scan is usually the first imaging method. Typical imaging features include an irregular shape, inhomogeneous density, calcification, high unenhanced CT attenuation values of more than 20 Hounsfield units, and inhomogeneous enhancement with intravenous contrast [9]. On magnetic resonance imaging (MRI), there is hypointensity compared with the liver on T1-weighted images, high to intermediate signal intensity on T2-weighted images, and a heterogeneous signal drop on chemical shift. A high standardized uptake value is seen in the FDG-PET-CT study. Evidence of local invasion or metastases can also be present. Evaluation for metastasis should include imaging of the following sites: liver, lungs, lymph nodes, and bone. The diagnosis is confirmed by histopathology [10].

The only curative approach to ACC is complete surgical resection via open surgery. Routine loco-regional lymphadenectomy, including periadrenal and renal hilum lymph nodes, is recommended. In addition, any suspicious or enlarged lymph nodes seen on preoperative imaging should also be removed [11]. For patients with hypercortisolism, hydrocortisone should be given perioperatively to prevent postoperative adrenal insufficiency. This is the rationale for administering hydrocortisone to our patient. Post-resection, patients should undergo surveillance with imaging every three months for three years, then every 3-6 months for three more years [10]. Adjuvant therapy with mitotane, an adrenocorticolytic drug, is still under study. It should be considered in patients without macroscopic residual tumor post-surgery but with a high risk of recurrence. If the decision is made to start mitotane, it is recommended to start post-resection of the tumor as soon as clinically possible. We deemed our patient a candidate for mitotane as pathology revealed a high proliferative index with a Ki-67 >30%. Another option for patients with advanced disease is immunotherapy with immune checkpoint inhibitors. Additional supportive therapy includes medical therapy for hormone excess, anti-resorptive medications for bone metastasis, palliative radiation for advanced or metastatic disease, and fertility counseling for female patients of reproductive age [12].

## Conclusions

We want to highlight the importance of recognizing that pathologic hormone secretion from rare endocrinologic tumors can present nonspecifically. We presented a case of ACC with isolated hypercortisolism causing hypertension, electrolyte derangements, and edema, which can masquerade as hyperaldosteronism or other common cardiac, renal, or autoimmune conditions leading to diagnostic challenges. Our case underscores the importance of maintaining a high index of suspicion for adrenal carcinoma in patients presenting with hypertension and hypokalemia, especially when traditional hormonal evaluation yields inconclusive results. Considering the unfavorable prognosis of this aggressive neoplasm, it is imperative to pursue further studies promptly to prevent delayed diagnosis, which could exacerbate morbidity and mortality. By elucidating the intricacies of this unusual presentation, we aim to contribute to the broader understanding of adrenal carcinoma and facilitate improved diagnostic and therapeutic strategies for similar cases in the future.
